# Improvement of Aspects of Subjective Sleep Quality of Healthy Volunteers by Ingestion of Porcine Placental Extract: A Randomized Cross-Over Pilot Study

**DOI:** 10.3389/fnut.2020.550287

**Published:** 2020-10-20

**Authors:** Mahiko Nagase, Chiharu Watanabe, Masataka Kitanohara, Masashi Nishiya, Takao Okada, Masami Ohishi, Yasuhiko Komatsu

**Affiliations:** ^1^Kichijoji Traditional Chinese Medicine Clinic, Tokyo, Japan; ^2^Department of Medical Education, Juntendo University Graduate School of Medicine, Tokyo, Japan; ^3^Chiharu Dermatology Clinic, Saitama, Japan; ^4^Kitanohara Women's Clinic, Miyagi, Japan; ^5^Hibikinomori Clinic, Hokkaido, Japan; ^6^Snowden Co., Ltd., Tokyo, Japan

**Keywords:** sleep, porcine placental extract, dietary supplement, Pittsburgh Sleep Quality Index, St. Mary's Hospital Sleep Questionnaire

## Abstract

**Objectives:** This study assessed the effects of oral porcine placental extract (PPE) on sleep quality of healthy volunteers not satisfied with their sleep.

**Design:** This study used a randomized, placebo-controlled, double-blind, cross-over clinical pilot study.

**Setting:** This study was conducted under an outpatient multicenter setting in Japan.

**Interventions:** A total of 20 healthy Japanese volunteers aged between 28 and 73, whose Pittsburgh Sleep Quality Index global scores were between 6 and 10, successfully completed the study. At first, PPE at 300 mg/kg or placebo was ingested for 2 weeks. Then, after a 2-week washout period, each group ingested under a cross-over setting the opposite sample (placebo or PPE) for another 2 weeks.

**Main Outcome Measures:** Objective measurement of the sleep made with an activity tracker and subjective measurements of sleep quality by use of St. Mary's Hospital Sleep Questionnaire were done just before and after the administration time slots.

**Results:** No effect of PPE on the sleep length was observed. Several measures in the subjective St. Mary's Hospital Sleep Questionnaire, i.e., changes in Q5 (sleep depth) and Q9 (sleep wellness) between pre- and post-ingestions, were significantly different between groups in the direction of improvement of subjective sleep quality in the PPE group.

**Conclusions:** Although oral PPE at 300 mg/day for 2 weeks did not affect the length of sleep itself, it significantly improved several measures of subjective sleep quality. These results suggest that PPE might be a way to improve sleep quality without hypnotic drugs.

**Clinical Trial Registration:** www.umin.ac.jp/ctr/, identifier: UMIN000026468.

## Introduction

Getting sufficient sleep is crucial for a healthy life of all people. Especially in the modern world, insufficient sleep has become recognized as the cause of a variety of health problems. For example, sleep deprivation causes excess daytime sleepiness and trouble in concentration, resulting in lower performance of tasks (1) as well as accidents (2, 3). Furthermore, insufficient sleep also affects the autonomic activity (4) which possibly leads to depression (5), diabetes (6), hypertension (7), atherosclerosis (7), and so on. Therefore, improvement of sleep is important to prevent such a variety of health troubles. However, because hypnotic drugs have inherent risks of side effects, other ways to improve sleep are needed.

One such way is taking food materials having a property of improving sleep. In this present study, we focused on porcine placental extract (PPE) as the food material having the potential to improve sleep. Recent studies on PPE showed that symptoms associated with menopausal women could be improved by the ingestion of PPE (8, 9). Because menopausal symptoms include sleep problems, we thought that sleep problems are potential target to be improved by PPE ingestion. Moreover, because studies on the effects of PPE in menopausal women reported that PPE did not affect the hormonal levels of the subjects (8, 9), PPE might also be effective in non-menopausal women and also in men. However, no such results have been reported to date.

In this pilot clinical study to evaluate the appropriateness of such a possibility as the basis of future definitive trial, we employed healthy subjects without medication for sleep problems but who were not satisfied with their sleep. PPE or placebo was ingested under a cross-over setting for 2 weeks, and sleep before and after each ingestion period was assessed by both objective and subjective ways.

## Materials and Methods

### Study Design and Subjects

This was a multicenter, randomized, double-blind, placebo-controlled pilot study under a cross-over setting conducted between March 2017 and July 2018. The study was done in compliance with the Declaration of Helsinki and was approved by the institutional review board of the Japan Society of Clinical Placenta Medicine. All subjects provided written informed consent before participating in the study. This study was registered with the University Hospital Medical Information Network Clinical Trials Registry (UMIN000026468).

Recruiting of subjects had been started at 7 outpatient clinics, and finally a total of 32 Japanese including clinics staffs were successfully recruited from among 4 of these clinics. The eligibility criteria were the following: (1) no satisfaction with sleep quality but no underlying disease; (2) agreement to participate in the study and to afford written informed consent; (3) not pregnant nor possibly pregnant; (4) not diagnosed as having any sleep disorder; (5) not having medical care for sleep disorder; (6) not using drugs/foods affecting sleep; (7) not using placental extract–containing drugs/foods; (8) Pittsburgh Sleep Quality Index (PSQI) global score of between 6 and 10; (9) age of 20 years or older; (10) not inappropriate as a subject by the decision of the doctor in attendance. The 32 subjects fulfilling the aforementioned criteria were permuted-block randomized 1:1 to either the group ingesting PPE (300 mg/day) or to one taking a placebo at first (named PPE-first and Placebo-first groups, respectively). The randomization was performed secretly to both doctors and subjects by the registration center at the Japan Society of Clinical Placenta Medicine.

**Figure 1:**
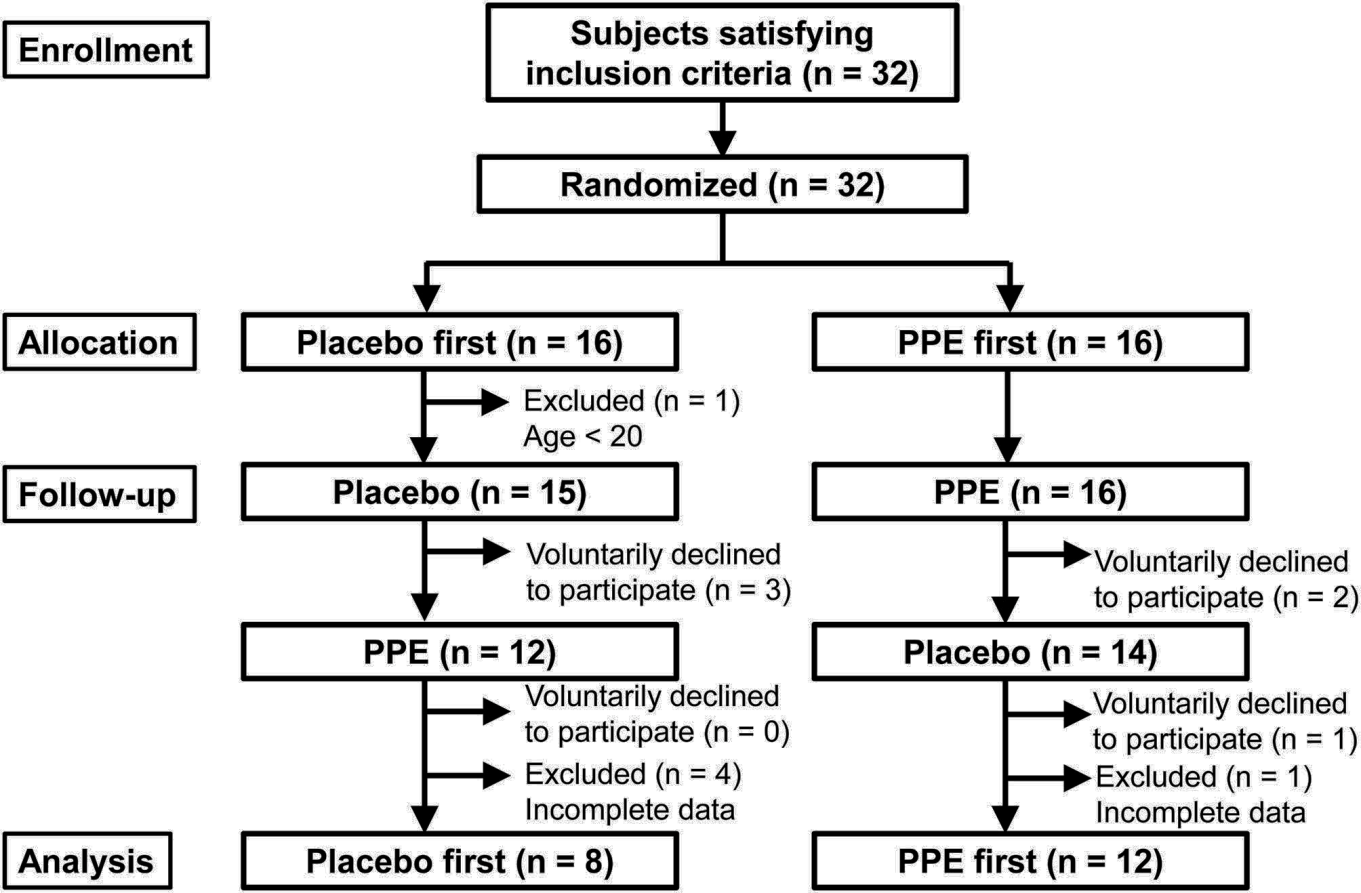
Flow diagram of the study.

After randomization, 5 and 1 subjects were excluded from PPE-first and Placebo-first groups, respectively, because of erroneous enrollment of a teenager (17 years old; *n* = 1) or incomplete data acquisition including one shift worker (*n* = 5), as shown in Figure 1. Furthermore, a total of 6 subjects (*n* = 3 in Placebo-first and *n* = 3 in PPE-first groups, respectively) voluntarily discontinued the study (Figure 1). Finally, 20 subjects completed the study and were used for data analyses. Because effects of glycine (10, 11) and l-serine (12) to improve sleep had been reported with *n* = 7 to 15, we thought *n* = 20 was sufficient for this pilot clinical study. To support this thought, we roughly estimated the necessary number of subjects using “power.t.test” running on R statistical software (13) based on the result about l-serine (12), in which 7 subjects were analyzed under cross-over setting with St. Mary's Hospital Sleep Questionnaire (SMHSQ) employed in this study (see next section). By supposing a type 1 error probability of 5% and a power of 80%, we got the numbers 7 and 6 for “Sleep well or not” and “Sleep satisfaction,” respectively, which were statistically significant in their report. Moreover, numbers 11, 11, and 10 were calculated for “Sleep depth,” “How clear-headed,” and “Difficulty in getting off to sleep,” respectively, suggesting *n* = 20 is a reasonable number for this study. However, because this estimation does not take “multiple number of measurements” into account, problems derived from such a multiplicity will be considered in Discussion section.

**Figure 2:**
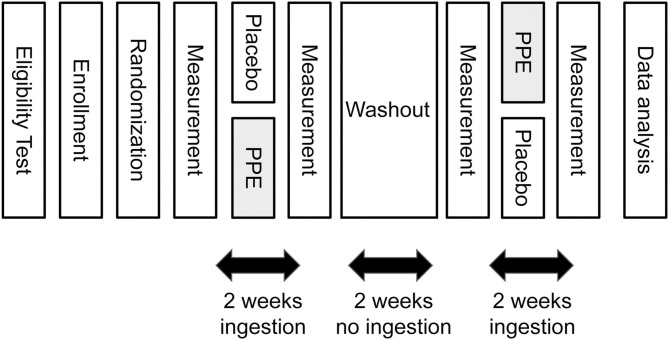
Time schedule of the study.

The outline of the time schedule of the study is depicted in Figure 2. One day before starting the ingestion of test samples, subjective and objective measurements of sleep were done as described later. Then the first round of daily 2 weeks' ingestion of PPE or placebo was started. At the last night of the ingestion period, the measurements of sleep were done again. Then a 2-week washout period followed, in which the subjects were instructed not to ingest test samples. At the last night of the washout period, the third measurements of sleep were done, and then the second round of 2 weeks' ingestion was commenced under the cross-over setting. Finally, at the last night of the second round of ingestion, the final measurements of sleep were conducted.

### Test Samples

The test samples (both PPE and placebo) used in this study were the same soft capsules reported previously (9). Because one PPE capsule contains 75 mg PPE, the dose of PPE 300 mg/day could be attained by the ingestion of 4 capsules/day. As PPE, Snowden's PPE powder (Snowden, Japan) produced by hydrolysis of porcine placenta with protease was used.

### Measurements

To evaluate the sleep state of possible participants beforehand, we employed PSQI (14), which is a self-rated questionnaire consisting of 19 questions about the sleep quality of the past 1 month. Because originally a PSQI global score of >5 was proposed as the discrimination point between good and poor sleepers (14), we used PSQI global score = 6 as the lower limit for the potential participants. As the upper limit, we set PSQI global score = 10, based on the reported mean PSQI global score for patients with insomnia (14) or disorders of initiating and maintaining sleep (15) being 12.1 or 10.4, respectively.

For objective measurement of sleep to evaluate the effect of PPE, we employed a wristwatch-type activity tracker (PULSENSE PS-500; Seiko Epson, Nagano, Japan). According to the manufacturer, sleep length of the subject could be calculated with the manufacturer's original software, in which not only total sleep length but also the ratio of deep and light sleeps is determined.

For subjective measurement of sleep to evaluate the effect of PPE, we employed SMHSQ (16), which consists of 14 questions related to sleep (see **Table 2**). According to Leigh et al. (17), three additional variables were calculated and used for analysis: sleep latency = time between Q1 and Q2; sleep period time = time between Q2 and Q3; awake onset latency = time between Q3 and Q4.

### Statistical Analysis

Significance of differences between pre- and post-treatment and between PPE and placebo groups were determined by performing the Wilcoxon signed-rank test by using R statistical software (13). Significance of differences between Placebo-first and PPE-first groups about baseline characteristics of the participants were determined by performing the Wilcoxon rank-sum test by using KaleidaGraph ver4.5 software. We considered it statistically significant when significance probability *p* < 0.05.

## Results

### Background Characteristics of Participants

Table 1 shows the background characteristics of the participants. A total of 32 potential subjects were recruited and randomized to either the PPE-first or Placebo-first group, and 25 subjects completed the ingestion and measurements (Figure 1). Because the data obtained from 5 participants, who completed the test, had been found missing, finally 20 participants were subjected to data analysis. As shown in Table 1, no significant differences were detected between PPE-first and Placebo-first groups regarding age or PSQI global score at either screening or analysis.

**Table 1:** Baseline characteristics of participants.

	**Placebo-first group**	**PPE-first group**	*P*-value^*^	**Total**
**(1) At screening**				
**Number of participants**				
Total	16	16		32
Female	16	14		30
Male	0	2		2
Age (years) (Range)	39.0 ± 10.8 (17–52)	48.2 ± 13.9 (26–73)	0.126	43.6 ± 13.1 (17–73)
Pittsburgh Sleep Quality Index global score (Range)	8.44 ± 1.21 (7–10)	8.13 ± 1.31 (6–10)	0.646	8.28 ± 1.25 (6–10)
**(2) At analysis**				
**Number of participants**				
Total	8	12		20
Female	8	11		19
Male	0	1		1
Age (years) (Range)	44.8 ± 6.8 (34–52)	50.3 ± 13.5 (28–73)	0.283	48.1 ± 11.4 (28–73)
Pittsburgh Sleep Quality Index global score (Range)	8.50 ± 1.20 (7–10)	8.08 ± 1.24 (6–10)	0.910	8.25 ± 1.21 (6–10)

* *Wilcoxon's rank sum test*.

### Effect of PPE on Objective Sleep Values

The data on sleep length taken from the activity tracker are depicted in Table 2, where in addition to total sleep length, lengths of deep and light sleep periods are also shown. Furthermore, using these data, we calculated the ratios of deep and light sleeps to the total sleep. As shown in Table 2, no statistical significances were detected between pre- and post-treatments or between placebo and PPE groups.

**Table 2:** Result of activity tracker and St. Mary's Hospital Sleep Questionnaire.

	**Placebo**				**PPE**				***P*-value^**b**^ (Post–Pre difference) (Placebo vs. PPE)**
	**Pre^**a**^**	**Post^**a**^**	***P*-value^**b**^ (Pre vs. Post)**	**Post–Pre^**a**^**	**Pre^**a**^**	**Post^**a**^**	***P*-value^**b**^ (Pre vs. Post)**	**Post–Pre^**a**^**	
**Activity Tracker**									
Deep Sleep (min)	110 ± 124	105 ± 113	0.766	−8.14 ± 127	153 ± 104	140 ± 100	0.383	−13.1 ± 84.9	0.784
Light Sleep (min)	257 ± 130	281 ± 113	0.409	21.5 ± 169	233 ± 99.8	236 ± 111	0.956	3.30 ± 123	0.648
Total Sleep (min)	368 ± 105	386 ± 70.1	0.330	13.4 ± 119	386 ± 86.2	376 ± 90.4	0.622	−9.80 ± 124	0.409
Deep Sleep%	27.8 ± 30.7	26.5 ± 26.6	0.799	−1.36 ± 32.8	38.4 ± 24.7	36.4 ± 25.0	0.729	−1.98 ± 21.0	0.756
Light Sleep%	72.2 ± 30.7	73.5 ± 26.6	0.799	1.36 ± 32.8	61.6 ± 24.7	63.6 ± 25.0	0.729	1.98 ± 21.0	0.756
**The St. Mary's Hospital Sleep Questionnaire**									
Sleep Latency (Q1 to Q2) (min)	46.0 ± 52.5	33.3 ± 21.5	0.279	−12.7 ± 48.2	55.1 ± 36.7	41.3 ± 47.1	0.0803	−13.8 ± 41.6	0.930
Sleep period time (Q2 to Q3) (min)	364 ± 92.9	363 ± 76.5	0.936	−0.350 ± 100.5	349 ± 73.1	376 ± 58.2	0.0933	26.8 ± 69.9	0.234
Awake onset latency (Q3 to Q4) (min)	25.0 ± 27.7	17.6 ± 18.5	0.307	−7.35 ± 25.5	24.0 ± 30.7	25.1 ± 24.0	0.738	1.15 ± 35.3	0.368
Q5 Sleep depth	4.35 ± 1.93	3.90 ± 1.71	0.447	−0.450 ± 2.21	3.75 ± 1.59	4.90 ± 1.48	**0.00952**	1.15 ± 1.79	**0.0116**
Q6 Wake up frequency	1.70 ± 1.34	1.70 ± 1.26	0.773	0.00 ± 1.26	1.55 ± 1.19	1.00 ± 1.21	0.146	−0.550 ± 1.50	0.252
Q7 How much sleep (min)	364 ± 93.6	367 ± 73.5	0.677	3.15 ± 97.2	351 ± 58.0	378 ± 61.9	0.0560	27.7 ± 57.9	0.337
Q8 Napping (min)	6.25 ± 18.4	5.3 ± 10.7	1.00	−1.00 ± 21.4	9.00 ± 40.2	13.5 ± 31.1	0.406	4.50 ± 53.3	0.609
Q9 Sleep well or bad	3.95 ± 1.39	3.80 ± 1.20	0.641	−0.150 ± 1.53	3.20 ± 1.32	4.35 ± 1.18	**0.00397**	1.15 ± 1.53	**0.0170**
Q10 How clear-headed	2.80 ± 1.47	2.90 ± 1.25	0.781	0.100 ± 1.41	2.40 ± 0.995	3.05 ± 1.32	0.0371	0.650 ± 1.23	0.221
Q11 Sleep satisfaction	2.85 ± 1.31	2.95 ± 1.05	0.827	0.100 ± 1.12	2.45 ± 0.945	3.45 ± 1.23	**0.00684**	1.000 ± 1.45	0.068
Q12 Trouble by early waking	1.10 ± 0.308	1.25 ± 0.444	0.250	0.150 ± 0.366	1.10 ± 0.308	1.10 ± 0.308	1.00	0.00 ± 0.324	0.375
Q13 Difficulty in getting off to sleep	1.70 ± 0.801	1.65 ± 0.875	0.797	−0.0500 ± 0.999	1.85 ± 0.875	1.40 ± 0.681	0.0566	−0.450 ± 0.887	0.339
Q14 Time to fall asleep (min)	38.8 ± 39.8	36.1 ± 28.3	0.938	−2.65 ± 38.7	51.6 ± 41.8	39.8 ± 51.6	0.156	−11.8 ± 49.8	0.397

a *The values are described as mean ± S.D*.

b *Statistical significances are determined with Wilcoxon signed rank test*.

*P-values < 0.05 are indicated by bold letters*.

### Effect of PPE on Subjective Sleep Values

Totally 13 subjective sleep data obtained from SMHSQ are also depicted in Table 2. As shown there, amounts of change associated with the ingestion of test samples (values of Post–Pre) were significantly different between placebo and PPE groups with respect to Q5 and Q9 (Wilcoxon signed-rank test). Regarding the differences between pre- and post-treatment, in addition to Q5 and Q9, sleep satisfaction (Q11) was also significantly improved only in the PPE group, although not significant between placebo and PPE groups. On the other hand, “sleep-period time (Q2 to Q3)” and “how much sleep (Q7)” were not significantly different between pre- and post-treatments or between placebo and PPE groups. These results taken together suggest that PPE ingestion for 2 weeks improved subjective sleep depth, sleep wellness, and sleep satisfaction, but did not change the sleep length itself.

## Discussion

In this study, we for the first time demonstrated with a pilot clinical study that 2-week daily ingestion of PPE (300 mg/day) significantly improved aspects of subjective sleep quality of healthy subjects who were not satisfied with their sleep compared with that of the placebo. The subjective sleep parameter values improved by PPE ingestion were “sleep depth (Q5)” and “sleep wellness (Q9),” which were significantly different between PPE and placebo groups (Table 2). Also, “sleep satisfaction (Q11)” was improved in the PPE group, but not in the placebo one, after the 2 weeks' ingestion (Table 2). However, no parameters related to sleep lengths were significantly affected by ingestion of PPE irrespective of the methods employed (objective and subjective). In addition to sleep length itself, the “ratio of deep sleep” determined by activity tracker, “sleep latency (Q1 to Q2),” “awake onset latency (Q3 to Q4),” “napping (Q8),” and “time to fall asleep (Q14)” determined by SMHSQ were not significantly changed by PPE (Table 2), suggesting that the placental extract did not affect any sleep length–related aspects of sleep.

Regarding apparatuses objectively measuring sleep, actigraphy has been used and recognized as a reliable tool for sleep measurement (18, 19). In this study, however, we tried to use the inexpensive consumer-use activity tracker, instead of actigraphy, but failed to show a positive effect of PPE on objective sleep quality. It is not clear at this time that PPE truly could not improve objective sleep quality because unfortunately, this apparatus could calculate only “total sleep” and the ratio of “deep sleep” to “total sleep.”

Regarding the evaluation of subjective sleep properties, SMHSQ (16) was used in the studies on glycine (10, 11) and l-serine (12), where positive effects were detected. Glycine improved “how clear-headed (Q10),” “Sleep satisfaction (Q11),” “Difficulty in getting off to sleep (Q13),” and “time to fall asleep (Q14)” (10, 11); whereas l-serine improved “Sleep wellness (Q9)” and “Sleep satisfaction (Q11)” (12). Because in our present study PPE improved Q5, Q9, and Q11, the effect of PPE resembled that of l-serine more than that of glycine. However, the l-serine contained in PPE cannot fully explain the effect of PPE of improving sleep quality because the effective dose of l-serine (3 g/day) (12) is extensively higher than that of PPE (300 mg/day), in which the free l-serine content was small.

Regarding study design, we employed 2 weeks' washout period in this pilot clinical study under cross-over setting, although Koike et al. (8) reported that the effect of PPE in climacteric women continued until 4 weeks after the cessation of its ingestion. However, because in their study PPE was daily ingested for 24 weeks compared with our 2 weeks before its cessation, their result cannot straightforwardly be applied to our study condition. Also, if the effect of PPE continued after the 2-week washout period, its effect on sleep in this study would have been underestimated under this cross-over setting because the effect of PPE possibly continued until placebo-ingestion period (PPE-first group). Therefore, 2 weeks' washout period could not affect the positive effect of PPE in this study.

In a previous study (9), we reported that ingestion of PPE improved the subjective health condition of climacteric women with weak menopausal symptoms, which condition was measured by use of a questionnaire. However, no effects of PPE on objectively assessed hormonal conditions were observed (9). In the present study, PPE successfully improved the subjective feeling of sleep quality without having any effect on objective sleep length. Based on these results, some yet unknown ingredients in PPE might act centrally in a direct or indirect manner to alleviate the feeling of unwellness in the case of menopausal symptoms and sleep, even if their objective aspects are not improved. Elucidation of such responsible factors in PPE and the underlying mechanism are challenges for the future.

In this study, we employed 15 different measurements (2 objective and 13 subjective ones), which inherently raises the problem of multiplicity in statistics. However, in the present paper, we did not take this problem into consideration because the main purpose of this study was to obtain information about target parameters to be confirmed in future definitive clinical study. As a result, the present study suggests that “sleep depth (Q5)” and/or “sleep wellness (Q9)” in SMHSQ will be the appropriate target(s) for the upcoming definitive clinical study. However, if multiple comparison is strictly applied, all the statistical significances in Table 2 disappear. This is a limitation of this pilot clinical study.

There exist other limitations in this study, including (1) a high proportion of participants withdrawn from the study (6 in 32), (2) a high proportion of missing data (5 in 32), (3) no systematic analysis of adverse events (AEs), and (4) inappropriate balance of gender. Therefore, future definitive studies should be done with larger number of participants with balanced gender, employing systematic analysis of AEs, and with the design making participants easy to continue the study. Concerning objective measurement of sleep, actigraphy instead of activity tracker will be appropriate as the apparatus to measure objective sleep quality. Moreover, to obtain more reliable data about sleep, it would be appropriate to measure five to seven consecutive daily sleeps instead of one (20).

## Data Availability Statement

The data used in this article are not publicly available because of participants' privacy concerns, but are available on reasonable request.

## Ethics Statement

The studies involving human participants were reviewed and approved by Institutional review board of the Japan Society of Clinical Placenta Medicine. The patients/participants provided their written informed consent to participate in this study.

## Author Contributions

CW, MK, and MNi collected data, contributed to discussion, and reviewed the article. MNa collected data, contributed to discussion, and wrote the article. YK contributed to discussion, analyzed data, and wrote the article. MO contributed to discussion, analyzed data, and reviewed the article. TO contributed to discussion and reviewed the article. YK was the guarantor of this work and took responsibility for the integrity of the data and the accuracy of the data analysis. All authors contributed to the design of the study and had full access to the data used in the study.

## Conflict of Interest

MO and YK were employed by Snowden Co., Ltd. The remaining authors declare that the research was conducted in the absence of any commercial or financial relationships that could be construed as a potential conflict of interest.
